# Systematic Review: Effect of Health Education Intervention on Improving Knowledge, Attitudes and Practices of Adolescents on Malnutrition

**DOI:** 10.3390/nu12082426

**Published:** 2020-08-13

**Authors:** Ruth Charles Shapu, Suriani Ismail, Norliza Ahmad, Poh Ying Lim, Ibrahim Abubakar Njodi

**Affiliations:** 1 Department of Community Health, Faculty of Medicine and Health Science, Universiti Putra Malaysia, Serdang 43400, Selangor, Malaysia; ruthyshapu52@gmail.com (R.C.S.); si_suriani@upm.edu.my (S.I.); pohying_my@upm.edu.my (P.Y.L.); 2 College of Nursing and Midwifery, Damboa Road, Maiduguri, Maiduguri 600252, Borno State, Nigeria; 3 Department of Physical and Health Education, University of Maiduguri, Maiduguri 600230, Borno State, Nigeria; ibrahimnjodi@gmail.com

**Keywords:** adolescents, knowledge, attitude, practice, malnutrition, intervention, health education, nutrition, undernutrition, risk of bias

## Abstract

Adolescence is a phase in the life cycle of human beings. Adequate knowledge, attitudes and practices towards malnutrition are necessary for proper growth and development and for their future children. This systematic review aimed to determine the effect of health education intervention to improve the knowledge, attitudes and practices of adolescents on malnutrition. PubMed, Scopus, clinical trials, CINAHL, SAGE, Science Direct and Medline were searched according to Preferred Reporting Item for Systematic Reviews and Meat-analysis (PRISMA) guidelines to identified published studies from January 2013 to December 2019 based on the inclusion and exclusion criteria. A total of eight studies were included in this review. Data extraction was done based on randomized controlled trial only. Three out of the eight studies had low risk of bias, the overall evidence of the study was moderate. Findings from this study suggest that health education intervention among adolescents have significantly improved their knowledge, attitudes and practices. More specific interventions should be conducted in low and middle income countries since they bear more of the burden of malnutrition globally.

## 1. Introduction

Adolescents are young individuals aged between 10 and 19 years old. Globally, there are 1.8 billion adolescents, constituting the largest generation of young persons and about 90% of them reside in low and middle income countries (LMIC). Adolescence, when growth spurts occur, may expose them to malnutrition [1,2,3,4,5,6]. They gain 20% to 25% of their height and up to 50% of their ideal weight [7,8]. To support this rapid growth, there is a need for increased demand of energy, protein, minerals and vitamins [1,9,10,11]. Sufficient nutrient intake of both macro and micronutrients is essential at this stage to meet the increased demand due to speedy growth, sexual maturation and menstruation [12,13]. Undernutrition may contribute to underweight, poor performance at school, poor general health, pregnancy and birth complications, and less economic productivity [14,15]. Whereas overnutrition may contribute to non-communicable diseases such as hypertension, coronary heart disease, stroke, diabetes, sleep apnea and cancer, among others [11,16].

Globally undernutrition deficiency is a risk factor contributing to the burden of disease among adolescents [17]. The prevalence of iron deficiency among younger adolescent girls in lower social development index countries is 25%, and 27% among older adolescent girls [18]. The prevalence of iodine deficiency is common among younger and older adolescent girls in lower social development index countries with prevalence rates of 3.4% and 4.6%, respectively. Insufficient iodine contributes to the burden of micronutrient deficiencies among adolescent girls [18]. The prevalence of vitamin deficiency in lower social development index countries is 20% among younger adolescent girls and 18% among older adolescent girls [18]. Physical growth among adolescents is a key indicator of their health status, the prevalence of underweight among adolescent and children (5 to 19 years old) is 8.4% for girls and 2.4% for boys [19]. A global school-based student health survey reveals that 4% of girls 13 to 15 years old are underweight. More than 10% of adolescent girls in Vietnam, Bangladesh, Mauritius, Maldives, Sudan and Cambodia are underweight [18]. There was limited information on stunting among adolescents especially girls; the prevalence of stunting among adolescent girls in Guatemala is 52%, Brazil 6%, Bangladesh 44%, and Kenya 8%, respectively [17]. The growth of adolescents is a window of opportunity for intervention aimed at improving their health and nutritional status in reducing the burden of malnutrition among these age groups [5].

Globally, the prevalence of overweight has increased from less than 1% in 1975 to 5% among girls and 8% among boys in 2016, while the prevalence of obesity has increased in all the regions of the world, with the largest increase of about 400% per decade from 1975 in southern Africa [5,19]. The trend of over-nutrition amplified from 5 million in 1975 to 50 million in 2016 among girls, and 6 million in 1975 to 74 million in 2016 among boys globally [19]. Undernutrition and overnutrition among adolescents in over 200 countries from 1975 to 2016 were found to be over 31 million. This shows the accelerating increase in the burden of malnutrition globally [8].

Adolescence also is a unique point of intervention as people of this age group are more receptive to changes in lifestyle that may determine their life course later [4]. Previous studies showed that adolescents have poor knowledge, attitudes and practices about malnutrition and dietary intake [10,14,20,21]. Providing them with knowledge about malnutrition could prevent them from malnutrition and related illnesses later in life. Studies have recommended the provision of health education and behavioral change intervention in order to prevent and reduce malnutrition among adolescents [22]. Such interventions include information on food groups, food diversity, food sources rich in micronutrients, hygiene, sanitation and consequences of malnutrition in later life [23,24,25]. Enhancing health education interventions on knowledge, attitudes and practices, especially among adolescents, is potentially important in reducing malnutrition and mitigating short and long term consequences associated with health outcomes and those of their future offspring. There is a dearth of information on the evaluation of study characteristics and the overall quality of evidence of intervention studies in improving knowledge, attitudes and practices among adolescents. This systematic review aimed to determine the effectiveness of health education intervention on improving knowledge, attitudes and practices of adolescents on malnutrition.

## 2. Materials and Methods

### 2.1. Search Method

This systematic review used the Preferred Reporting Item for Systematic review and Meta-analysis guidelines (PRISMA) [18]. A search was conducted on electronic data bases. These include PubMed, Scopus, Clinical Trials, CINAHL, SAGE, EMBASE and Medline between January 2013 and December 2019 to review the latest health education intervention on knowledge, attitudes and practices of adolescents on malnutrition. Including the most recent health education intervention in this study will serve as a guide for intervention studies in this contemporary dispensation whereby the study protocols are in line with changing trends in both behavior and lifestyle. The search process was conducted using the following keywords: (health education OR nutrition education OR nutrition information OR dietary information OR school based intervention OR intervention) AND (knowledge OR attitude OR practice) AND (adolescents OR teenagers OR children OR students OR secondary school children) AND (Nutrition OR Malnutrition OR under nutrition OR inadequate dietary intake OR over nutrition OR obese). The study protocol was registered in PROSPERO (international database of prospectively registered systematic reviews) under the study identification code CRD42019128882.

### 2.2. Eligibility Criteria

Eligibility criteria were according to PICOS (population, intervention, comparison, outcome, and study design).

**Population:**  Adolescents age 10 to 19 years old with a minimum study population of 50. Study population below the age of 10 years and above 19 years were excluded from the study.
**Intervention:**  Interventions on nutrition, healthy eating/diet, dietary intake, anemia, fruits and vegetables were included in the study.
**Comparison:**  A comparison group with no intervention or given other trial interventions were included. Studies with no direct comparison group, such as quasi experimental studies, were excluded from the study.
**Outcome:**  Studies with knowledge or attitude or practice as the study outcome were included.
**Study design:**  Randomized controlled trials/clustered randomized control trials were included in the study. Studies with quasi experimental studies, non-randomized controlled trials, or the study design not mentioned were excluded.

Other report characteristics include (i) Studies in English language, (ii) Studies published between January 2013 and December 2019, were included. Studies excluded include (i) studies with irrelevant title not related to research topic, (ii) studies not available (only abstract), (iii) literature reviews or systemic reviews, (iv) qualitative study, (v) books, conference proceedings, reports, (vi) thesis, (vii) case studies.

### 2.3. Data Extraction

**Figure 1:**
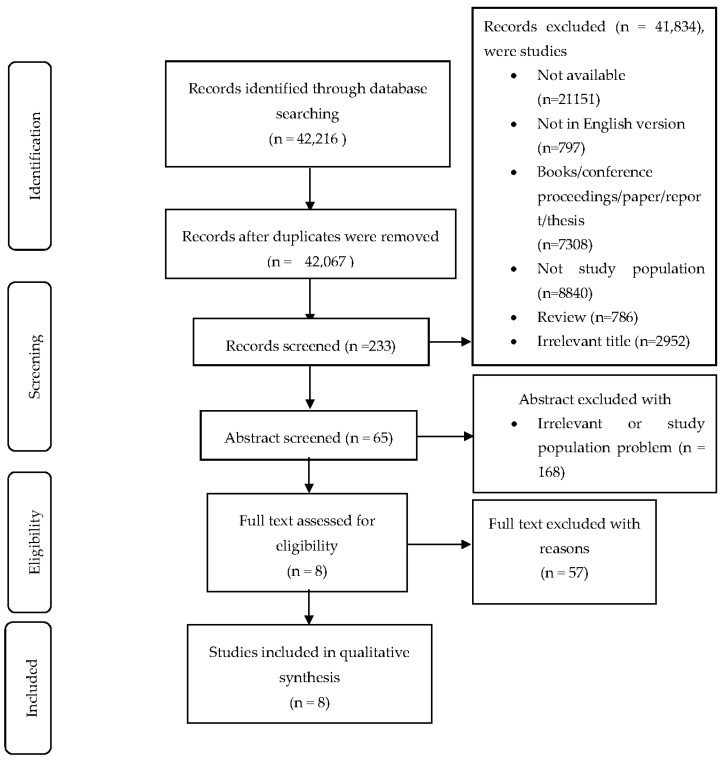
Preferred Reporting Item for Systematic Review and Meta-analysis (PRISMA) 2009, four phase flow diagram of literature search [19].

The Preferred Reporting Item for Systematic Review and Meta-analysis (PRISMA) diagram was used to summarize the search item presented in Figure 1. A total of 42,216 were identified, with 41,834 excluded because they were duplicates, conference proceedings, had irrelevant titles, were not available in the English language, did not have the correct study population, or were reviews. Out of the 233 articles retrieved, 168 were excluded based on the abstract. A total of 65 studies were further assessed and 57 studies were excluded based on age group, not being a randomized controlled trial, the study design not being mentioned, the outcome not aiming at improving knowledge or attitude or practice, the studies not being on health or nutrition education intervention, and not being related to malnutrition. A total of eight studies were included in the final analysis. Studies were screened by two independent reviewers (R.C.S and L.P.Y) and disagreements among the reviewers were resolved by mediators (S.B.I, N.A and I.A.N).

### 2.4. Quality Assessment of the Study

Two assessment tools, including (1) Cochrane Collaboration Risk of Bias Tool, and (2) Grading of Recommendations, Assessment, Development and Evaluation (GRADE) were used to evaluate all the intervention studies. The two assessment tools are described below.

#### 2.4.1. Risk of Bias

The Cochrane Risk of Bias Assessment Tool was used to evaluate the types of bias in each of the studies presented in supplementary Table S1. The Cochrane Collaboration Risk of Bias Tool scale contains 12 items, which assess the internal and external validity of studies. The review evaluated and rated the 12 items. Items rated ‘yes’ were scored as ‘1′, while no or unable to determine or unclear or non-applicable were all scored as ‘0′. Higher scores and percentages indicate a lower risk of bias. The level of bias within each category for each study was rated as ‘high risk’ or ‘low risk’. Each criterion had equal weight, or the same value; the total score was calculated as the percentage of the maximum value obtained. Studies with scores above the mean score were considered to have a low risk of bias, while studies below the mean value are considered to have a high risk of bias [26,27,28,29].

#### 2.4.2. GRADE Quality Assessment

GRADE is a systematic approach used in developing and presenting summaries on the quality of evidence across studies and thus helps in making recommendations [30]. The quality of evidence is evaluated based on the type of evidence, quality points, consistency, directness and effect size [30,31]. The final score of at least four points indicates high quality of evidence, three points reflects moderate quality of evidence, two points suggests low quality of evidence, and one point represents a very low quality of evidence. 

### 2.5. Intervention Intensity

Intervention intensity was used to identify its association with the effectiveness of the intervention. The intensity of each intervention was represented as high or medium or low intensity, adopted from recent reviews [32,33]. The intervention was assessed on a five (5) point Likert scale ranging from (1 = low, 2 = low medium, 3 = medium, 4 = medium high, 5 = high) based on four characteristics of the intervention [32,33]:

1. The duration of the intervention. This shows the length of the intervention (1 = ≤6 weeks, 2 = 6 to 11 weeks, 3 = 12 weeks to 5 months, 4 = 6 to 12 months, 5 = ≥12 months).
2. The frequency of contact with the intervention. This assessed the frequency of contact between the intervention and the participants. In cases where the intervention used more than one contact, the average score of contact was calculated. The score on frequency of contact ranges from (1 = annually, 2 = bimonthly to quarterly, 3 = monthly, 4 = weekly, 5 = daily).
3. Type of contact (level of personalization). This assessed the type and the level of contact with the intervention. 1 = environmental, 2 = adolescent only, 3 = group (adolescents and teachers), 4 = group (adolescents and parents), 5 = group (adolescents and parents and teachers and community).
4. The reach of the intervention strategies. This assessed the different settings used where 1 = one setting, 3 = two settings and 5 = three or more settings. The larger the number of settings in the intervention, the greater the intensity of the intervention.

The total intensity score of the intervention was based on the four characteristics that sum up the overall intensity score of 20. The total intervention intensity score of ≤10.5 was considered as low intervention intensity, 10.51 to 13.49 was considered as medium intervention intensity, ≥13.5 was considered as high intervention intensity [33].

## 3. Results

### 3.1. Search Findings

#### 3.1.1. Study Characteristics

The eight studies were summarized based on authors/year of study/country/study design, study settings/duration of intervention, target population/theory used, description of the intervention, outcome assessed/statistical significance of the outcome, effect size, and effectiveness of the intervention (Table 1).

#### 3.1.2. Country and Study Design

Out of the eight studies included, four were from high income countries [35,36,39,40], two from middle income countries [37,38], and two from low income countries [34,41]. Three of the studies were individual randomized controlled trials [34,36,41] and five were clustered randomized controlled trials [35,37,38,39,40].

#### 3.1.3. Setting and Target Population

From the eight studies reviewed, five studies were school-based interventions involving only the adolescents [35,36,37,38,41]; where three studies were curriculum interventions (incorporated into the school curriculum) [37,38], two were non-curriculum interventions, which were offered as after school programs [35,36], one was a school-based intervention but did not mention whether it was a curriculum-based intervention or non-curriculum intervention [41]. Two were community-based interventions [34,39], and one study was conducted in a recreational center, which included some kind of family and community involvement [40]. Four studies targeted only girls [34,38,39,41], and four studies targeted both boys and girls [35,36,37,40].

#### 3.1.4. Duration, Approaches and Contents of Intervention

The duration of the intervention varied significantly from three weeks [39], five weeks [36], three months [35,41], six months [37], eight months [38,40] and two years [34]. Only one study had a follow up of 8–12 weeks [39].

The health education intervention included the following approaches and contents: (i.) Personal or group discussion [34]. (ii.) Peer leaders, club/peer support activities and social marketing [36,37]. (iii.) In-class components, school components, family/community components [37,38]. (iv.) Observational modelling, persuasive communication and active learning [39]. (v.) Taste test, cooking demonstration [35,36,40]. (vi.) Physical activities [35,38]. (vii.) Videos, and lectures [34,35,36,37,38,39,40,41]. Topics included food groups, food sources, food pyramid, balanced diet, nutrition deficiency, food safety, dietary diversity, hygiene and sanitation, fruits and vegetables, sport nutrition, and physical activities [34,35,36,37,38,39,40,41].

#### 3.1.5. Sample Size, Control Group, Effect Size, Attrition Rate

The sample size for the intervention studies ranged from small groups of adolescents (*n* = 89 each) [39,41], (*n* = 130) [37], to large groups of adolescents (*n* = 432, *n* = 500, *n* = 1784, *n* = 2279, *n* = 4022) [34,35,36,38,40] that participated in the intervention program. Seven studies did not mention activities or programs provided to control groups [34,36,37,38,39,40,41]. Only one study mentioned a normal after school activity (homework, arts and crafts) given by their teacher after school hours to the control group [35]. Only one intervention study provided information on calculated effect size [38]. Four out of the eight studies mentioned the inclusion of attrition rate in the sample size calculation as 10% [38,41], and 20% [35,39].

#### 3.1.6. The Use of Theory

Two out of the eight studies reviewed defined a behavioral theory for the intervention i.e., diffusion innovation theory [36] and theory of planned behavior [39]. However, there was no explanation on how the constructs of these theories were used in the studies. The remaining six studies were not theory-based studies [34,35,37,38,40,41].

#### 3.1.7. Intervention Intensity

The overall rating categories of intervention intensity are presented in Table 2. In this review, four studies were rated as low intensity [34,35,39,41], two studies were rated as medium intensity [36,37], two studies were rated as high intensity [38,40].

Three studies with low intensity were effective with statistically significant (*p* < 0.05) improvement in knowledge, attitudes and practices among adolescent girls [34,41,42]. Only one study with low intervention intensity was ineffective with *p* > 0.05 [35]. The remaining studies with medium intervention intensity [36,37,43] and high intervention intensity reported statistically significant (*p* < 0.05) improvement in knowledge, attitudes and practices [38,40].

The duration of the intervention varied significantly from three weeks [35], five weeks [33], three months [32,37], six months [40], eight months [36,39] and two years [38]. Only one study had a follow up of 8–12 weeks [35].

The frequency of contact was not mentioned in two studies [34,38]. Monthly participant contact was effective in one study [40] but was not effective in another study [35]. All the remaining studies with the frequency of contact ranging from annually, bi-monthly to quarterly, weekly and daily were effective [36,37,39,40,41].

The level of personalization with only adolescent girls was not effective in one study [35] but was effective in the remaining three studies [34,39,41]. Studies that involved groups of either adolescents and teachers [36,37] or adolescents and parents [40] or adolescents, teachers and parents [38] were all effective in improving knowledge, attitudes and practices of adolescents.

Studies that involved only one setting were effective in improving knowledge, attitudes and practices in five studies [34,35,37,39,41] out of five studies. Two studies that involved two settings [36,40] and a study with three or more settings demonstrated significant improvement in knowledge, attitudes and practices of adolescents towards malnutrition [38].

### 3.2. Quality of Reporting

#### 3.2.1. Risk of Bias

All the eight intervention studies included in this review were assessed for risk of bias [34,35,36,37,38,39,40,41]. The summary of the risk of bias by authors and the judgment of each risk of bias item were presented in Table 3. The average score of the eight studies reviewed was 5.1; studies with total score ≥ 5.1 were considered low risk of bias. Three studies were rated with low risk of bias [35,38,39], while the remaining five studies were rated as studies with high risk of bias [34,36,37,40,41]. Four studies mentioned the reliability of the study instruments [35,37,39,40]. Seven studies had participant retention rates of ≥70% [34,35,36,37,38,39,41].

#### 3.2.2. Grades of Recommendation, Assessment, Development and Evaluation (GRADE)

All the eight studies were included in the GRADE analysis [34,35,36,37,38,39,40,41]. The summary of the individual studies was presented in Table 4. The quality of evidence based on GRADE in Abdur Razzak et al. (2016) was low, with a low quality point (no follow up and did not mention blinding, allocation concealment and attrition rate), moderate consistency and directness, and did not mention effect size [34]. Lachausse (2017) had moderate quality of evidence, moderate quality point (no follow up, did not mention blinding and allocation concealment and had a response rate of more than 70%), moderate consistency and directness and did not mention effect size [35]. Bogart et al. (2014) had moderate quality of evidence, with moderate quality point (no follow up, mentioned blinding, did not mention allocation concealment and attrition rate), moderate consistency, high quality of directness but did not mention effect size [36]. Wang et al. (2015) had low quality of evidence, with low quality point (no follow up, did not mention blinding, allocation concealment and attrition rate), moderate quality of consistency and directness and did not mention effect size [37]. Saraf et al. (2014) had high quality of evidence, moderate quality point (no follow up, mentioned blinding, did not mention allocation concealment and had response rate of more than 70%), moderate quality of the use of effect size, high quality consistency and directness [38]. Laram et al. (2017) had moderate quality of evidence, moderate quality point (there was a follow up, did not mention blinding and allocation concealment, the study had a response rate of >70% and a population of less than 200), consistency and directness with very low quality of the use of effect size [39]. Shin et al. (2015) showed low quality of evidence, low quality point (no follow up, did not mention blinding and allocation concealment, the study had a response rate of <70% and had a population of less than 200), moderate quality of consistency and directness, high quality of the use of effect size [40]. Jalambo et al. (2017) revealed low quality of evidence, very low quality point (no follow up, did not mention blinding and allocation concealment, the study revealed a response rate of >70% and had a population of less than 200), moderate consistency and directness and very low quality of the use of effect size [41].

The overall quality of evidence across the eight studies was moderate with an overall score of three [34,35,36,37,38,39,40,41]. All studies received a score 4 as all the studies reviewed were RCT studies. The quality point was low. The consistency of the overall intervention evidence was moderate with seven out of the eight studies being effective.

### 3.3. Effect of the Intervention

#### 3.3.1. Knowledge

Seven [34,36,37,39,40,41] out of the eight studies reported statistically significant effects in improving knowledge, as presented in Table 2. There were significant changes in knowledge on malnutrition, food groups, food types, food pyramid, good sources of iron, food preparation, balanced diet, healthy food purchasing, healthy beverages, healthy snacks, iron deficiency anemia, fruit, and vegetables, from baseline to post intervention [34,35,36,37,38,39,40,41] and follow up [39] among the intervention group.

#### 3.3.2. Attitude

The impact of the health education and nutrition education intervention was statistically significant in five studies [34,36,37,39,41]. These included attitude towards importance of nutrition to health, importance of developing healthy dietary habits, food with an expired date, heme-iron intake and vitamin C, willingness to protect themselves against iron deficiency anemia, eating food, cafeteria attitude, drinking tap water, voluntary skipping of meals and avoiding meat products [34,36,37,39,41] Table 2.

#### 3.3.3. Practice

Seven [34,36,37,39,40,41] out of the eight studies had statistically significant improvement from the outcome of the health and nutrition education intervention. These included practice towards improved meal frequency, taking of iron tablets, fruit and vegetable consumption, healthy eating, dietary diversity and adequate water consumption [34,36,37,39,41].

## 4. Discussion

The review was intended to identify the characteristics of the effect of health and nutrition education intervention aimed at improving knowledge, attitudes and practices on malnutrition among adolescents. The review systematically identified and summarized the characteristics of the effect of health/nutrition education intervention in improving knowledge, attitudes and practices of adolescents on malnutrition [34,35,36,37,38,39,40,41].

Seven studies (87.5%) included in the review reported effectiveness of the intervention in increasing knowledge, attitudes and practices (*p* < 0.05) among adolescents, either through school-based intervention or community-based intervention or using both school and community-based intervention. This was in line with studies conducted in China and Italy [44,45,46].

Evidence exists that the use of theory in developing health education modules with effective learning skills effectively improved knowledge, attitudes and practices, thereby possibly decreasing the risk factors associated with pre-adolescent and adolescent malnutrition [46,47,48,49]. One study reported ineffectiveness resulting from low quality of the intervention delivery that led to low response from participants. The ineffectiveness of the intervention can be associated with the absence of an underlying theory of behavioral change. Studies have suggested that effective interventions that are theory-based provide opportunities for participants to learn and practice skills through interactive teaching, role play, videos, play competition and demonstration aimed at changing their behavioral practice [50,51,52].

Interventions to improve knowledge, attitudes, practices and the nutritional status of adolescent have significantly improved nutritional status and healthy behaviors among adolescents. However, it must be noted that most of the studies (six out of eight) (*n* = 6) were centered in developed countries (high income countries) limiting the generalizability since most of the findings were from high income countries. It is necessary to implement intervention that has evidence significance in low and middle income countries that have a higher burden of malnutrition, food security and hygiene, with a strong focus on the health and nutritional needs of adolescent girls. Health education intervention on malnutrition will improve the health status of adolescents now, when they become adults, and for the optimal growth and development of their offspring to prevent the cycle of intergenerational transmission of malnutrition in adolescents. The effectiveness of the intervention in this review concurs with evidence from systematic reviews that showed statistically significant improvement in knowledge, attitudes and practices [53,54,55,56]. The effectiveness of the intervention in this review also concurs with other health education interventions on the knowledge, attitudes and practices regarding physical health, psychosocial health and reproductive health among adolescents in India, Saudi Arabia and Zimbabwe [57,58,59].

Seven out of nine studies reviewed were school-based interventions targeting adolescent girls that are in schools, indicating the scarcity of community-based intervention among adolescent girls; this is in line with previous studies targeting school settings [26]. More studies should include community-based intervention to target the marginalized adolescents who are not in school, either through the youth centers, the community clinics and the compound of the community leader/stakeholder. Intervention to reduce the risk of malnutrition should be implemented through the community platforms in low and middle income countries since addressing the burden of malnutrition is indirectly related to poverty, environmental sanitation and hygiene, infection, poor education and literacy [60,61,62].

Intervention intensity analysis was used to relate the effectiveness of intervention. Two interventions targeting adolescents, parents, teachers and the community recorded the highest intervention intensity scores and all demonstrated effectiveness, while four interventions targeting only adolescents had low intervention intensity scores, with three out of the four studies being effective and one being ineffective. This was similar to a study conducted in Tunisia, Italy, Greece, Australia and Brazil, which reveals that studies of adolescents with family members and teachers inclusive were effective in improving knowledge, attitudes and practices towards physical education, health makers, school food aid and social cognitive mediators [46,63,64,65,66]. In line with this, the study in Michigan involving adolescents, teachers, and the state board of education was effective in improving student dietary intake [67]. The results from this review show that there is a strong parental influence on adolescents. There is need to support the inclusion of parents and adolescents in interventions aiming to increase their knowledge, attitudes and practices. Considering the characteristics of intervention intensity, there was no evident association between intervention duration or frequency of contact and overall effectiveness of intervention. In contrast, the level of personalization and the reach of intervention (intervention settings) show an association with overall effectiveness of intervention.

Most of the studies have sufficient participant retention rates ranging from 73% to 96% [34,35,36,37,38,39,41], with only one having a retention rate below 70% [40]. Only one study used intention to treat analysis [35], and five studies used subjective reporting [34,35,37,38,39,41], while the three remaining studies used both objective and subjective tools such as a stadiometer and electronic scale [36,40,41], one study used blood parameters but the instrument and procedures were not mentioned in the study [41]. Two studies did not mention about seeking parental consent. The principle of respect requires parents to exercise voluntary choice in their child’s participation in any research [68]. High risk of bias was reported in 62.5% of the studies reviewed. This concurs with a systematic review that reported 50% high risk of bias [33].

The overall quality of evidence based on GRADE criteria was moderate. This demonstrates the reliability of the overall intervention process and conclusion. This suggests the need to (i) improve the standards and procedures in intervention design and outcome reporting in randomized controlled trials to support the identification of important outcomes that are relevant to the study population. (ii) Iidentify the outcomes that have the tendency of improving the effectiveness of health/nutrition education interventions. (iii) To enable the comparison of the methodology of the study in order to determine the factors that promote the effectiveness of health/nutrition education interventions among adolescents. Improving on the methodological quality, such as random sequence generators, allocation concealment, blinding of participants, management of how drop out, the inclusion of objective reporting, and follow up after intervention, will increase the quality of the study and also the overall study outcome.

More context-specific and relevant studies should be conducted in middle and low income countries since these settings bear more burden of malnutrition globally and early marriage, especially among adolescent girls, is high in these settings. Most adolescent girls in low and middle income countries go into motherhood with little or no knowledge about malnutrition and its consequences; focusing on adolescent girls is not only important for her but also for her children in the near future in preventing the cycle of intergenerational transmission of malnutrition. Furthermore, most of the interventions targeted both adolescent boys and girls; intervention studies should be gender sensitive in some settings to help close the gap that exists within these age groups.

The limitations of the review include limited available information on malnutrition-specific interventions among adolescents in low and middle income countries, no specific intervention studies addressing pregnant adolescents within the age range of 10 to 19 years, lack of intervention studies targeting adolescent girls from minority populations, limited intervention studies designed for community-based settings to target adolescent girls that are not in school, and there were no intervention studies targeting married adolescent girls. Randomized controlled trials should have post-intervention follow-ups to ensure the sustainability of the intervention. Future studies should include post-intervention follow-ups in their study protocol. Furthermore, future studies should focus on underprivileged low and middle income populations either through school-based or community-based interventions.

## 5. Conclusions

Health and nutrition education interventions to improve knowledge, attitudes and practices on malnutrition demonstrated variable success. Three out of eight studies had low risk of bias, and the overall evidence of the review was moderate. The evidence presented in this review has identified characteristics that may contribute to the effectiveness of interventions in increasing knowledge, attitudes and practices of adolescents towards malnutrition. The overall evidence from this review reveals the need to improve the standards and procedures in intervention design, in randomized controlled trials, to improve their effectiveness.

## Figures and Tables

**Table 1:** Summary of intervention to improve knowledge, attitudes and practices among adolescents on malnutrition.

Author, Year, Country, Study Design	Settings/Duration/Frequency of Intervention	Target Population/Theory	Description of the Intervention (I: Intervention, C: Control)	Outcome Assessed, Significance	Effect Size ^2^	Effective (Y/N) ^1^
Abdur Razzak et al., 2016 [34] Bangladesh, RCT ^3^.	Community-based/ 2 years/NM	10 to 19 years old girls (*n* = 250) No theory	I: Nutrition education was communicated through group or personal discussion (malnutrition, dietary diversity, food taboos, hygiene and sanitation) to adolescent girls using charts, leaflets, posters.	Knowledge (*p* < 0.001) Attitude (*p* < 0.05) Practice (*p* < 0.05) Anthropometric (*p* < 0.05) Reported pre and post-test intervention changes in nutritional status.	ND	Y
			C: No action was provided to control group			
Lachausse, 2017 [35] USA, CRCT ^4^	School-based (after school program)/ 3 months/Monthly	Grade 4 to 6 boys and girls, (*n* = 275) No theory	I: Harvest of the month (HOTM) nutrition education program on fruits and vegetables consumption, and physical activities including fruits and vegetable tasting, students work book, nutritional information presentations, story books, farm to school presentation, HOTM newsletter for parents, menu slicks, and cafeteria posters	Knowledge (*p* > 0.05) Self-efficacy (*p* > 0.05) Reported pre and post-test on fruit and vegetable consumption, knowledge, and self-efficacy on fruit and vegetable consumption	ND	N
			C: Normal after school activity (homework tutorial, arts and crafts) as assigned by their after school teacher.			
Bogart et al., 2014 [36], USA, RCT ^3^	School-based/ 5 weeks/daily	Grade 7 boys and girls, (*n* = 4022) Diffusion of innovation theory	I: Students for Nutrition and eXercise intervention (SNaX), including school food environment changes, peer leader club and social marketing (cafeteria food taste test, nutritional messages, and educational book marks)	Knowledge (*p* < 0.01) Attitude (*p* < 0.05) Intention (*p* < 0.05) Reported pre-test and post-test on cafeteria food tasting, knowledge on healthy eating/physical activity, and intention	ND	Y
			C: No action was provided to control group			
Wang et al., 2015 [37], China, CRCT ^4^	School-based/ 6 months/weekly	Grade 7 to 9 boys and girls, (*n* = 130) No theory	I: Nutrition education intervention including in-class nutrition curriculum, peer support activities and the distribution of brochures using mass media, television (TV) messages, information leaflets.	Knowledge (*p* < 0.05) Attitude (*p* < 0.05) Healthy eating behaviour (*p* < 0.05) Reported pre-test and post-test on knowledge, attitude and healthy eating behaviour	ND	Y
			C: No action was provided to control group			
Saraf et al., 2014 [38], India, CRCT ^4^	School-based/ 8 months/NM	Grade 6 and 7 girls (*n* = 2279) No theory	I: Health education on diet, physical activities and tobacco through school component, class room component and family/community component using health education lectures, flash films, peer group discussion, flip charts, physical training (PT) classes	Knowledge (*p* < 0.01) Behavioural practice on physical activity, diet and tobacco (*p* < 0.01) Reported pre-test and post-test for knowledge and behavioural practices	39%	Y
			C: No action was provided to control group			
Laram et al., 2017 [39], Canada, CRCT ^4^	Community-based/ 3 weeks/Weekly	12 to 17 years old girls, (*n* = 89) Theory of planned behaviour (TPB)	I: Nutrition education on healthy eating and sport nutrition through persuasive communication, active learning, observational modelling, using lectures, brainstorming, and discussion	Knowledge (*p* < 0.001) Attitude (*p* < 0.001) Subjective norm (*p* < 0.01) Intention (*p* > 0.05) Perceived behavioural control (*P* > 0.05)	ND	Y
			C: No action was provided to control group			
Shin et al., 2015 [40], USA, CRCT ^4^	Recreation centre/ 8 months/daily	10 to 14 years’ boys and girls, (*n* = 152) No theory	I: Nutrition education: The Baltimore Healthy Eating Zones (BHEZ) intervention in recreation centres (corner stores/carryout restaurants and food outlets), with a focus on healthy eating, beverages, breakfast, snacks, and cooking at home through activities such as lectures, taste tests, cooking demonstrations, shelf labels, point of purchase, posters and flyers	Knowledge (*p* < 0.001) Behavioural intention (healthy food purchase, beverages, snacks, and food preparation) (*p* = 0.01) Outcome expectancy (*p* = 0.02) Self-efficacy (*p* = 0.54) BMI (*p* < 0.04)	ND	Y
			C: No action was provided to control group			
Jalambo et al., 2017 [41], Palestine, RCT ^3^	School-based/ 3 months/weekly	15 to 19 years old girls, (*n* = 89) No theory	I: Nutrition education on food groups, food pyramid, balanced food, iron absorption enhancers and inhibitors, sources of iron, anaemia and iron deficiency using lectures, wall writing, videos, booklets and brochures	Knowledge (*p* < 0.001) Attitude (*p* < 0.001) Nutrition practice (*p* < = 0.002)	ND	Y
			C: No action was provided to control group			

^1^ Effectiveness of the intervention is defined as a statistically significant improvement in the study outcome (*p* < 0.05). ^2^ The abbreviation for ND is no data or data not available or not enough to calculate effect size. The abbreviation for RCT ^3^ is individual randomised control trial and CRCT ^4^ is cluster randomised control trial.

**Table 2:** Summary of overall intervention intensity.

Study (*n* = 9)	^1^ Duration	^2^ Frequency of Contact	^3^ Level of Personalization	^4^ Reach of the Intervention	Overall Intensity Score	^5^ Overall Intensity Rating	^6^ Effective
Abdur Razzak et al., 2016 [34] Bangladesh.	5	^7^ NM	2	1	8	Low	Y
Lachausse, 2017 [35] USA.	3	3	2	1	9	Low	N
Bogart et al., 2014 [36] USA.	1	5	3	3	12	Medium	Y
Wang et al., 2015 [37] China.	4	4	3	1	12	Medium	Y
Saraf et al., 2014 [38] India.	4	NM	5	5	14	High	Y
Laram et al., 2017 [39] Canada.	1	4	2	1	8	Low	Y
Shin et al., 2015 [40] USA.	4	5	4	3	16	High	Y
Jalambo et al., 2017 [41] Palestine.	3	4	2	1	10	Low	Y

^1^ Duration: 1 = ≤6 weeks, 2 = 6 to 11 weeks, 3 = 12 weeks to 5 months, 4 = 6 to 12 months, 5 = ˃12 months. ^2^ Frequency of contact: 1 = annually, 2 = bi-monthly to quarterly, 3 = monthly, 4 = weekly, 5 = daily. ^3^ Level of personalization: 1 = environmental, 2 = adolescent only, 3 = group (adolescents and teachers), 4 = group (adolescents and parents), 5 = group (adolescents and parents and teachers and community). ^4^ Reach of intervention: 1 = one setting, 3 = two settings, 5 = three or more settings. ^5^ Overall intensity rating: low intensity = ≤10.5, medium intensity = 10.51 to 13.49, high intensity = ≥13.5. ^6^ Effectiveness of the intervention is defined by statistical significance of (*p* < 0.05). ^7^ NM = refers to not mentioned in the study.

**Table 3:** Risk of bias ratings for each study included.

Study	Item ^1^	Item ^2^	Item ^3^	Item ^4^	Item ^5^	Item ^6^	Item ^7^	Item ^8^	Item ^9^	Item ^10^	Item ^11^	Item ^12^	Total *n* (%)	Bias
Abdur Razzak et al. (2016) [34]	0	0	1	0	1	0	1	0	0	1	0	0	4 (33%)	High
Lachausse (2017) [35]	0	0	1	0	1	1	1	0	0	1	1	1	7 (58%)	Low
Bogart et al. (2014) [36]	0	0	0	1	1	0	1	1	0	1	0	0	5 (42%)	High
Wang et al. (2015) [37]	0	0	0	0	1	1	1	0	0	1	0	0	4 (33%)	High
Saraf et al. (2014) [38]	1	0	1	1	1	0	1	0	0	1	0	1	7 (58%)	Low
Laram et al. (2017) [39]	0	0	1	0	1	1	1	1	1	1	0	1	8 (67%)	Low
Shin et al. (2015) [40]	0	0	1	0	1	1	1	0	0	0	0	0	4 (33%)	High
Jalambo et al. (2017) [41]	0	0	1	0	1	0	1	0	0	1	0	1	5 (42%)	High

Note. ^1^ Random sequence generation, ^2^ allocation concealment, ^3^ participants characteristics, ^4^ blinding, ^5^ intervention description, ^6^ outcome measurement of validity and reliability, ^7^ selective reporting, ^8^ use of theory, ^9^ follow up, ^10^ participant’s retention, ^11^ intention to treat, ^12^ attrition. Items rated “yes” were scored as “1”, “no” or unable to determine or unclear were both scored as “0”. Higher scores and percentages indicate a lower risk of bias.

**Table 4:** GRADE result for the eight individual studies.

Study	Type of Evidence	Quality Point	Consistency	Directness	Effect Size	Quality of Evidence
Abdur Razzak et al. (2016) [34]	4	2	3	3	0	Low
Lachausse (2017) [35]	4	3	3	3	0	Moderate
Bogart et al. (2014) [36]	4	3	3	4	0	Moderate
Wang et al. (2015) [37]	4	1	3	3	0	Low
Saraf et al. (2014) [38]	4	3	4	4	3	High
Laram et al. (2017) [39]	4	3	3	3	0	Moderate
Shin et al. (2015) [40]	4	2	3	3	0	Low
Jalambo et al. (2017) [41]	4	1	3	3	0	Low
Overall Quality	High	Low	Moderate	Moderate	Very low	Moderate

## Supplementary Materials

The following are available online at https://www.mdpi.com/2072-6643/12/8/2426/s1, Table S1: Cochrane Collaboration Risk of Bias Tool scale item descriptions.

## References

[1] UNICEF (2011). The State of the World’s Children 2011-Executive Summary: Adolescence an Age of Opportunity.

[2] WHO (2015). The Global Strategy for Women’s and Children’s and Adolescents’ Health (2016–2030): Survive, Thrive, Transform.

[3] World Health Organization (2002). The Optimal Duration of Exclusive Breastfeeding: Report of an Expert Consultation: Geneva, Switzerland 28–30 March 2001.

[4] Bakrania S., Ghimire A., Balvin N. (2018). Bridging the Gap to Understand Effective Interventions for Adolescent Well-Being: An Evidence Gap Map on Protection, Participation, and Financial and Material Well-Being in Low-And Middle-Income Countries.

[5] Christian P., Smith E.R. (2018). Adolescent Undernutrition: Global Burden, Physiology, and Nutritional Risks. Ann. Nutr. Metab..

[6] UNFPA & UNICEF (2012). Fact Sheet: Girls and Young Women. United Nations Adolescent Girls Task Force.

[7] Ward S., Hisley S. (2015). Maternal-Child Nursing Care Optimizing Outcomes for Mothers, Children, and Families.

[8] World Health Organization (2018). Guideline: Implementing Effective Actions for Improving Adolescent Nutrition.

[9] Mokhtari F., Kazemi A., Soheila E. (2017). Effect of educational intervention program for parents on adolescents’nutritional behaviors in Isfahan in 2016. J. Educ. Health Promot..

[10] Blum R.W., Gates W.H. (2015). Girlhood, not Motherhood Preventing Adolescent Pregnancy.

[11] WHO (2005). Nutrition in Adolescence—Issues and Challenges for the Health Sector.

[12] Sireesha G., Rajani N., Bindu V. (2017). Teenage girls ’ knowledge attitude and practices on nutrition. Int. J. Home Sci..

[13] World Health organization(WHO) (2018). Adolescents: Agents of Change for a Well-Nourished World: An Expert Consultation on Nutrition Programming for the Next Generation. https://www.who.int/nutrition/events/2018-consultation-adolescents-19to29jun/en/.

[14] World Health Organization (2004). Adolescent Pregnancy: Issues in Adolescent Health and Development.

[15] World Health Organization Adolescent Nutrition: A Review of the Situation in Selected South-East Asian Countries (No. SEA-NUT-163).

[16] Branca F., Piwoz E., Schultink W., Sullivan L.M. (2015). Nutrition and health in women, children, and adolescent girls. Br. Med. J..

[17] Black R.E., Victora C.G., Walker S.P., Bhutta Z.A., Christian P., De Onis M., Ezzati M., Grantham-Mcgregor S., Katz J., Martorell R. (2013). Maternal and child undernutrition and overweight in low-income and middle-income countries. Lancet.

[18] Akseer N., Al-gashm S., Mehta S., Mokdad A., Bhutta Z.A. (2017). Global and regional trends in the nutritional status of young people: A critical and neglected age group. Ann. N. Y. Acad. Sci..

[19] (2017). NCD Risk Factor Collaboration (NCD-RisC) Worldwide trends in body-mass index, underweight, overweight, and obesity from 1975 to 2016: A pooled analysis of 2416 population-based measurement studies in 128·9 million children, adolescents, and adults. Lancet.

[20] Gupta A., Noronha J.A., Garg M. (2018). Dietary intake of macronutrients and micronutrients among adolescent girls: A cross sectional study. Clin. Epidemiol. Glob. Health.

[21] Mangiaterra V., Pendse R., Mcclure K., Rosen J., Mouli V., Camacho V., Mathai M., Portela A., Zupan J., Olukoya P. (2008). Adolescent Pregnancy. WHO MPS Notes.

[22] Nelima D. (2015). Prevalence and Determinants of Anaemia among Adolescent Girls in Secondary Schools in Yala Division Siaya District, Kenya. Univers. J. Food Nutr. Sci..

[23] Rosen J.E., Adolescent Health and Development (AHD), The World Bank 2004 Apr:281627-1095698140167. https://citeseerx.ist.psu.edu/viewdoc/download?doi=10.1.1.630.384&rep=rep1&type=pdf.

[24] Promoting Girls’ Nutrition in Early Adolescence. http://documents1.worldbank.org/curated/pt/505211566452421860/pdf/Promoting-Girl-s-Nutrition-in-Early-Adolescence-a-last-window-of-opportunity.pdf.

[25] Arora G., Kochar G.K. (2016). Impact of Nutrition Education on Knowledge, Attitude, Practices and Beliefs of Adolescent Girls Belonging to Rural and Urban Area of District Kurukshetra. Int. J. Nutr. Food Sci..

[26] Camacho-Miñano M.J., LaVoi N.M., Barr-Anderson D.J. (2011). Interventions to promote physical activity among young and adolescent girls: A systematic review. Health Educ. Res..

[27] Steiner G.Z., Mathersul D.C., Macmillan F., Camfield D.A., Klupp N.L., Seto S.W., Huang Y., Hohenberg M.I., Chang D.H. (2017). A Systematic Review of Intervention Studies Examining Nutritional and Herbal Therapies for Mild Cognitive Impairment and Dementia Using Neuroimaging Methods: Study Characteristics and Intervention Efficacy. Evid.-Based Complementary Altern. Med..

[28] Higgins J., Altman D., Sterne J. (2011). Assessing risk of bias in included studies: Cochrane review. Cochrane Collab..

[29] Reviews I. (2011). The Cochrane Public Health Group Guide for developing a Cochrane protocol. Cochrane Consum. Commun. Gr.

[30] Ryan R., Hill S. (2016). How to GRADE the quality of the evidence. Cochrane Consum. Commun. Gr..

[31] Rafiq M., Boccia S. (2018). Application of the GRADE Approach in the Development of Guidelines and Recommendations in Genomic Medicine. Cochrane Consum. Commun. Gr.

[32] Hendrie G.A., Brindal E., Baird D., Gardner C. (2013). Improving children’s dairy food and calcium intake: Can intervention work? A systematic review of the literature. Public Health Nutr..

[33] Srbely V., Janjua I., Buchholz A.C., Newton G. (2019). Interventions Aimed at Increasing Dairy and/or Calcium Consumption of Preschool-Aged Children: A Systematic Literature Review. Nutrients.

[34] Abdur Razzak M., Mahfuz Al Hasan S., Shahinur Rahman S., Asaduzzaman M., Matin Juliana F., Sabir Hossain M. (2016). Role of nutrition education in improving the nutritional status of adolescent girls in North West areas of Bangladesh. Int. J. Sci. Eng. Res..

[35] LaChausse R.G. (2017). A clustered randomized controlled trial to determine impacts of the Harvest of the Month program. Health Educ. Res..

[36] Bogart L.M., Cowgill B.O., Elliott M.N., Klein D.J., Hawes-Dawson J., Uyeda K., Elijah J., Binkle D.G., Schuster M.A. (2014). A Randomized Controlled Trial of Students for Nutrition and eXercise: A Community-Based Participatory Research Study. J. Adolesc. Heal..

[37] Wang D., Stewart D., Chang C., Shi Y. (2015). Effect of a school-based nutrition education program on adolescents’ nutrition-related knowledge, attitudes and behaviour in rural areas of China. Environ. Health Prev. Med..

[38] Saraf D.S., Gupta S.K., Pandav C.S. (2014). Effectiveness of a School Based Intervention for Prevention of Non-communicable Diseases in Middle School Children of Rural North India: A Randomized Controlled Trial. Indian J. Pediatrics.

[39] Laram C., Drapeau V., Valois P., Goulet C. (2017). Evaluation of a Theory-Based Intervention Aimed at Reducing Intention to Use Restrictive Dietary Behaviors Among Adolescent Female Athletes. J. Nutr. Educ. Behav..

[40] Shin A., Surkan P.J., Coutinho A.J., Suratkar S.R., Campbell R.K., Rowan M., Sharma S., Dennisuk L.A., Karlsen M., Gass A. (2015). Impact of Baltimore Healthy Eating Zones: An Environmental Intervention to Improve Diet Among African American Youth. Heal. Educ. Behav..

[41] Jalambo M.O., Sharif R., Naser I.A., Karim N.A. (2017). Improvement in Knowledge, Attitude and Practice of Iron Deficiency Anaemia among Iron-Deficient Female Adolescents after Nutritional Educational Intervention. Glob. J. Health Sci..

[42] Jacob R., Lamarche B., Provencher V., Laramée C., Valois P., Goulet C., Drapeau V. (2016). Evaluation of a Theory-Based Intervention Aimed at Improving Coaches’ Recommendations on Sports Nutrition to Their Athletes. J. Acad. Nutr. Diet..

[43] Wang D., Stewart D., Chang C. (2016). Original Article Is an ecological school-based nutrition intervention effective to improve adolescents’ nutrition-related knowledge, attitudes and behaviour in rural areas of China?. Global Health Promot..

[44] Zhou W., Xu X., Li G., Sharma M., Qie Y., Zhao Y. (2016). Effectiveness of a school-based nutrition and food safety education program among primary and junior high school students in Chongqing, China. Glob. Health Promot..

[45] Naghashpour M., Shakerinejad G., Lourizadeh M.R. (2014). Nutrition Education Based on Health Belief Model Improves Dietary Calcium Intake among Female Students of Junior High Schools. J. Health Popul. Nutr..

[46] Gallotta M.C., Iazzoni S., Emerenziani G.P., Meucci M., Migliaccio S., Guidetti L., Baldari C. (2016). Effects of combined physical education and nutritional programs on schoolchildren’s healthy habits. PeerJ.

[47] Kesten J.M., Griffiths P.L., Cameron N. (2011). A systematic review to determine the effectiveness of interventions designed to prevent overweight and obesity in pre-adolescent girls. Obes. Rev..

[48] Salam R.A., Hooda M., Das J.K., Arshad A., Lassi Z.S., Middleton P., Bhutta Z.A. (2016). Interventions to Improve Adolescent Nutrition: A Systematic Review and Meta-Analysis. J. Adolesc. Health.

[49] Keshani P., Kaveh M.H., Faghih S., Salehi M. (2019). Improving diet quality among adolescents, using health belief model in a collaborative learning context: A randomized field trial study. Health Educ. Res..

[50] Kropski J.A., Keckley P.H., Jensen G.L. (2008). School-based Obesity Prevention Programs: An Evidence-based Review. Behav. Psychol..

[51] Łuszczki E., Sobek G., Bartosiewicz A., Baran J., Weres A., Dereń K., Mazur A. (2019). Analysis of Fruit and Vegetable Consumption by Children in School Canteens Depending on Selected Sociodemographic Factors. Medicina.

[52] Kupolati M.D., Macintyre U.E., Gericke G.J. (2018). A Theory-Based Contextual Nutrition Education Manual Enhanced Nutrition Teaching Skill. Front. Public Health.

[53] Wan T.T.H., Rav-Marathe K., Marathe S. (2016). A Systematic Review on the KAP-O Framework for Diabetes Education and Research. Med. Res. Arch..

[54] Rabiu A., Simbak N.B., Haque M.A. (2014). Systematic Review of Knowledge, Attitude and Practice on Adverse Drug Reactions and Pharmacovigilance among Doctors. J. Appl. Pharm. Sci..

[55] Barzkar F., Baradaran H.R., Koohpayehzadeh J. (2017). Knowledge, Attitude, And Practice Of Evidence-Based Medicine Among Physicians: A Systematic Review. Biomed. J..

[56] Xu T., Tomokawa S., Gregorio E.R., Mannava P., Nagai M., Sobel H. (2020). School-based interventions to promote adolescent health: A systematic review in low- and middle-income countries of WHO Western Pacific Region. PLoS ONE.

[57] Kaushal P., Singh T., Padda A.S., Deepti S.S. (2018). Effectiveness of a health education intervention on the knowledge, attitude and practices of teachers regarding physical and psychosocial health of adolescents in Amritsar, Punjab. Int. J. Community Med. Public Health.

[58] Elsayed E., Elsabagh M. (2017). Effect of Health Education Intervention on Knowledge and Practice about reproductive health among Adolescent Female Students. J. Med. Sci. Clin. Res..

[59] Midzi N., Mtapuri S., Mutsaka M.J., Ruhanya V., Magwenzi M., Chin N., Nyandoro G., Marume A., Kumar N., Mduluza T. (2014). Impact of School Based Health Education on Knowledge, Attitude and Practice of Grade Three Primary School Children in Zimbabwe. J. Community Med. Health Educ..

[60] Adolescent Nutrition: Policy and programming in SUN+ countries. https://resourcecentre.savethechildren.net/node/8970/pdf/adolescent_nutrition.pdf.

[61] Sawyer S.M., Afifi R.A., Bearinger L.H., Blakemore S.J., Dick B., Ezeh A.C., Patton G.C. (2012). Adolescence: A foundation for future health. Lancet.

[62] Lassi Z.S., Moin A., Das J.K., Salam R.A., Bhutta Z.A. (2017). Systematic review on evidence-based adolescent nutrition interventions. Ann. N. Y. Acad. Sci..

[63] Maaloul J., Harrabi I., Delpierre C., Gaha R., Ghannem H. (2013). Predictors of food and physical activity patterns among schoolchildren in the region of Sousse, Tunisia. Obes. Res. Clin. Pract..

[64] Brito K., Fiaccone R.L., Couto R.D., Ribeiro-silva R.D.C. (2015). Evaluation of the effects of a programme promoting adequate and healthy eating on adolescent health markers: An interventional study. Nutr. Hosp..

[65] Zota D., Dalma A., Petralias A., Lykou A., Kastorini C.M., Yannakoulia M., Karnaki P., Belogianni K., Veloudaki A., Riza E. (2016). Promotion of healthy nutrition among students participating in a school food aid program: A randomized trial. Int. J. Public Health..

[66] Mccabe B.E., Plotnikoff R.C., Dewar D.L., Collins C.E., Lubans D.R. (2015). Social Cognitive Mediators of Dietary Behavior Change in Adolescent Girls. Am. J. Health Behav..

[67] Alaimo K., Oleksyk S.C., Drzal N.B., Golzynski D.L., Lucarelli J.F., Wen Y., Velie E.M. (2013). Effects of Changes in Lunch-Time Competitive Foods, Nutrition Practices, and Nutrition Policies on Low-Income Middle-School Children’s Diets. Child. Obes..

[68] Roth-Cline M., Nelson R.M. (2013). Parental permission and child assent in research on children. Yale J. Biol. Med..

