# Supplementary material for: Systematic Review: Effect of Health Education Intervention on Improving Knowledge, Attitudes and Practices of Adolescents on Malnutrition
Source: Nutrients. 2020 Aug 13;12(8):2426. doi: 10.3390/nu12082426 (PMC7469070; doi:10.3390/nu12082426)
Supplement: Supplementary file 1 [file nutrients-12-02426-s001.pdf]

Table 1: **Cochrane Collaboration Risk of Bias Tool scale item descriptions**

Risk of bias item	Label	Description
1	Random sequence generation	Was the allocation sequence adequately generated?
2	Allocation concealment	Was allocation adequately concealed?
3	Participant characteristics	Are the characteristics of the participants included in the study clearly described (inclusion/exclusion criteria)?
4	Blinding of participants, personnel, and outcome assessors	Was knowledge of the allocated intervention adequately prevented during the study?
5	Intervention description	Is the intervention of interest sufficiently described to allow replication?
6	Outcome measurement validity and reliability	Was there a description of the instrument reliability/validity (reference or coefficient) or did they use a well-established known valid measure?
7	Selective reporting	Were all outcome measures detailed in the methods reported in the results?
8	The use of theory	Did the study use any theory
9	Were the participants followed up (after post-test)?	Were participants followed up for a minimum of 2 months?
10	Was retention rate adequately described?	If yes, was retention rate ≥ 70% at post intervention or post intervention follow?
11	Was intention to treat analysis used	The use of intention to treat?
12	Reporting of power calculation and attrition rate effect on power	Was a power calculation adequately reported
